# Association of HSS score and mechanical alignment after primary TKA of patients suffering from constitutional varus knee that caused by combined deformities: a retrospective study

**DOI:** 10.1038/s41598-021-81285-6

**Published:** 2021-02-04

**Authors:** Zhifeng Zhang, Wei Chai, Guanghui Zhao, Qida Zhang, Zhenxian Chen, Xinyao Wang, Pingping Wei, Yanwei Zhang, Zhongmin Jin, Yusheng Qiu

**Affiliations:** 1 Department of Orthopaedics, The First Affiliated Hospital, Xi’an Jiaotong University, Xi’an, 710061 Shaanxi China; 2 Department of Orthopaedics, First Medical Center of PLA General Hospital, Beijing, China; 3 Department of Joint Surgery, The Second Affiliated Hospital, Inner Mongolia Medical University, Hohhot, 010030 Inner Mongolia China; 4 Hong-Hui Hospital, Xi’an Jiaotong University College of Medicine, Xi’an, 710054 Shaanxi China; 5 State Key Laboratory for Manufacturing System Engineering, School of Mechanical Engineering, Xi’an Jiaotong University, Xi’an, 710054 Shaanxi China; 6 Key Laboratory of Road Construction Technology and Equipment of MOE, Chang’an University, Xi’an, 710064 Shaanxi China; 7 School of Mechanical Engineering, Tribology Research Institute, Southwest Jiaotong University, Chengdu, 610031 Sichuan China; 8 Institute of Medical and Biological Engineering, School of Mechanical Engineering, University of Leeds, Leeds, LS2 9JT UK

**Keywords:** Diseases, Rheumatic diseases, Osteoarthritis

## Abstract

For pre-operative osteoarthritis (OA) patients with varus knee, previous studies showed inconsistent results. Therefore, we conducted this study to better identify the association of Hospital for Special Surgery (HSS) score and mechanical alignment. 44 patients (51 knees) with constitutional varus knee caused by combined deformities (LDFA (lateral distal femoral angle) > 90°and MPTA (medial proximal tibial angle) < 85°)) were selected and analyzed with a mean follow-up period of 14 months after total knee arthroplasty (TKA). From January 2015 to December 2016, patients were collected consecutively after primary TKA. After filtering, fifty-one knees (44patients) were analyzed with a mean follow-up period of 14 months. All patients were divided into two groups based on post-operative hip-knee-ankle (HKA) acute angle: varus mechanical alignment (VMA) group (HKA < − 3°) and neutral mechanical axis (NMA) group (− 3° ≤ HKA ≤ 3°). 30 knees were included in the NMA group, and 21 knees in the VMA group. Comparisons of HSS between NMA group and VMA group were performed. After adjusting for age and Body Mass Index (BMI) confounders, Compared with NMA group, the HSS score in VMA group decreased by 0.81 units (95% CI, − 3.37 to 1.75) *p* = 0.5370). For pre-operative constitutional varus knee caused by combined deformities in chinese populations, no significant association between post-operative lower limb mechanical alignment and HSS score was found.

## Introduction

Generally, achieving a post-operative neutral lower limb mechanical alignment within 3° is regarded as a golden standard in total knee arthroplasty (TKA) for a long time, which is widely regarded being associated with optimal clinical function. Mechanical alignment principle demands that tibial and femoral cuts are perpendicular to the mechanical axis of the tibia and femur in the coronal plane, respectively^1^. Based on this principle, the neutral mechanical alignment in the coronal plane is defined as or characterized by hip-knee-ankle (HKA) acute angle of 0° ± 3°(mean and standard deviation)^2–4^.

The Hospital for Special Surgery (HSS)score ^5^ which was proposed by American Hospital in 1976 consists of the following six parts: pain (30 points), function (22 points), range of motion (18points), muscle strength (10points), knee flexion deformity(10points), and stability (10points). When the patients walk with the help of the crutches or there are varus deformity or valgus deformity in their knee-joints, these points will be reduced accordingly. HSS scoring system^6^ emphasizes pain, function and range of motion, which is well known for its high degree of inter-observer correlation^7^. The clinical function score is a reflection of the satisfaction score. Dissatisfaction is the patients’ unsatisfied state or attitude; which consist of discontent; displeased, or a particular reason or feeling of displeasure or disappointment. It is estimated that 20% of elderly osteoarthritis patients (about 60 million people) in the United States will need surgery by 2020^8^ . However, about 20% of patients are not satisfied with clinical outcome of TKA ^9–11^. Therefore, even a small promotion of satisfaction could have a substantial impact on patients who are performed with TKA.

Constitutional varus knee was characterized by the physiologic mechanical axis is 3° varus or more^12^, which takes a big proportion in medial OA patients^13^. Although many studies have explored the association between post-operative mechanical alignment and clinical outcomes in pre-operative varus knee populations, it is worth noting that previous studies showed inconsistent results^14–16^. Some studies^14,16^ found that TKA performed in patients with pre-operative varus deformity had superior clinical outcomes when the lower limb mechanical alignment was left in residual varus, while another studies^17–19^ found that post-operative varus mechanical alignment has no significant effect on patient’s clinical outcomes compared with the neutral mechanical alignment. And the majority of patients in above studies were Europeans or Americans who previously had varus knees, and their results may not be generalized to other populations with different lifestyles or disease.

Therefore, we conducted a retrospective study in Chinese populations to better identify the association of post-operative mechanical alignment and HSS score after primary TKA of patients suffering from constitutional varus knee that caused by combined deformities.

## Methods

### Demographics

**Figure 1:**
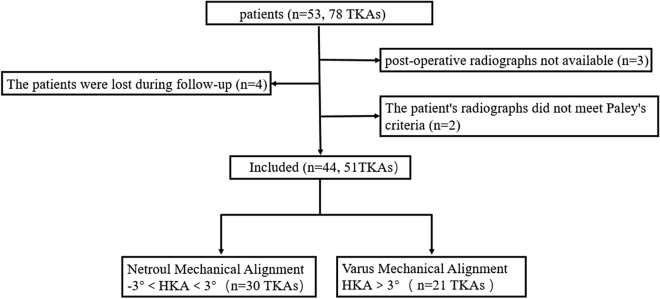
Flowchart of patients included in the study.

From January 2015 to December 2016, a doctor who was not involved in the surgery consecutively collected clinical data in this research. The target are patients suffering from constitutional varus knee that caused by combined femoral and tibial deformities before surgery ^12^ was consecutively collected. To get accurate experimental data, patients with bone on bone were excluded in this study. Each patient is selected according to the following inclusion and exclusion criteria. The inclusion criteria encompasses following points: (1) unilateral medial end-stage OA ((Kellgren-Lawrence grade) K/L grade III or IV) and contralateral knee joints with cartilage loss less than 50% (K/L grade 0–II); (2) bilateral medial end-stage OA patients (K/L grade III). The exclusion criteria includes five points: (1) rheumatoid or inflammatory arthritis; (2) external joint deformity; (3) posttraumatic; (4) abnormal condition caused by diseases (such as Paget’s or rickets etc.); (5) inappropriate preoperative or post-operative radiographs. 53 patients (78 knees) met above standard totally. After filtering, 44 patients (51 knees) who met inclusion criteria underwent follow-up (Fig. 1). 3 patients who met inclusion criteria could not participate in the experiment because post-operative radiographs were not available (n = 3). 4 patients were lost during follow-up (n = 4); 2 patient's radiographs did not meet Paley's criteria (n = 2). This study have got the informed consent of all patients. Ethical approval was granted by the Chinese PLA General Hospital medical ethics committee (S-2018-018-01). All experiments were performed in accordance with relevant guidelines and regulations.

### Radiographic evaluation

Pre-operative and post-operative full-length standing radiographs were collected and measured according to Paley’s criteria using the Mimics medical imaging programme (version 16.0; Materialise, Leuven, Belgium) with an accuracy within 0.1 mm^20^. All patients stood barefoot with the patellae pointed straight ahead^20^ during radiographic scan. The criteria of the femoral and tibial mechanical axis defined by Cooke et al^21^ was adopted in this study.

### LDFA

The LDFA is the lateral angle that formed by the mechanical axis of the femur and the knee joint line of the femur in the coronal plane.

### MPTA

The MPTA is the medial angle which formed by the mechanical axis of the tibia and the knee joint line of the tibia in the coronal plane.

### HKA

**Figure 2:**
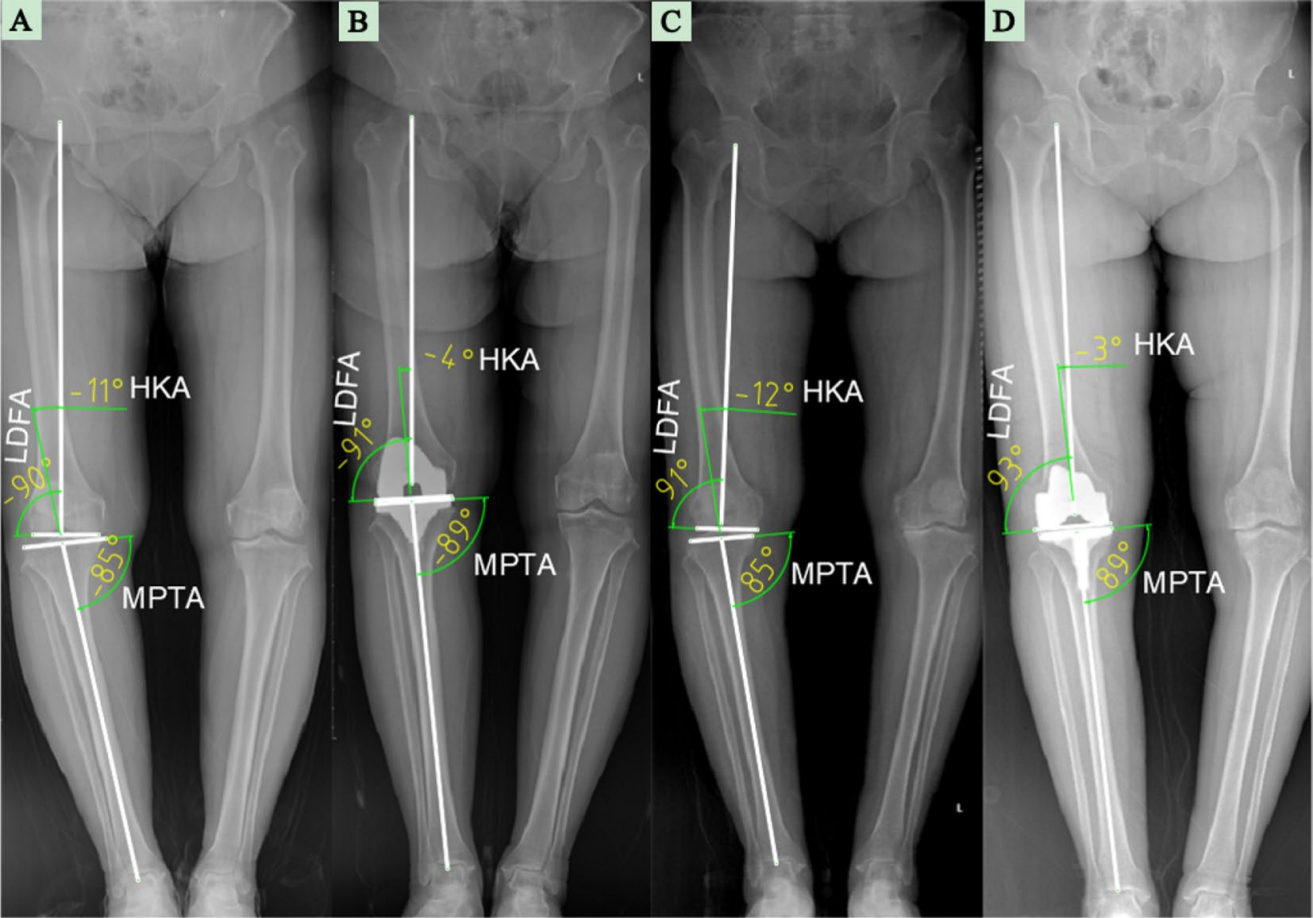
Radiographic measurements of HKA, LDFA and MPTA. Schematic diagrams of preoperative **(A)** and postoperative **(B)** standing radiograph for VMA groups, and preoperative **(C)** and postoperative **(D)** standing radiograph for NMA group.

The HKA angle was defined as the included angle between the femoral mechanical axis and the tibial mechanical axis^22,23^ (Fig. 2). This acute angle was expressed as a deviation from 0° with a negative value for varus knee and positive value for valgus knee.

### Clinical evaluation

Pre-operative and post-operative knee functional scores were calculated using the HSS scale^24^. The patients’ baseline characteristics included age, BMI and follow-up time. The maximum range of motion of the knee was measured by a goniometer at 12 months after surgery.

### Statistical analysis

Comparative statistical analyses between VMA group and NMA group after TKA were performed in SPSS software 22.0 (SPSS, Chicago, IL) with the independent sample t test, a *p* value < 0.05 was adopted. These measured parameters were summarized by the mean and standard deviation.

## Results

### General observations

**Figure 3:**
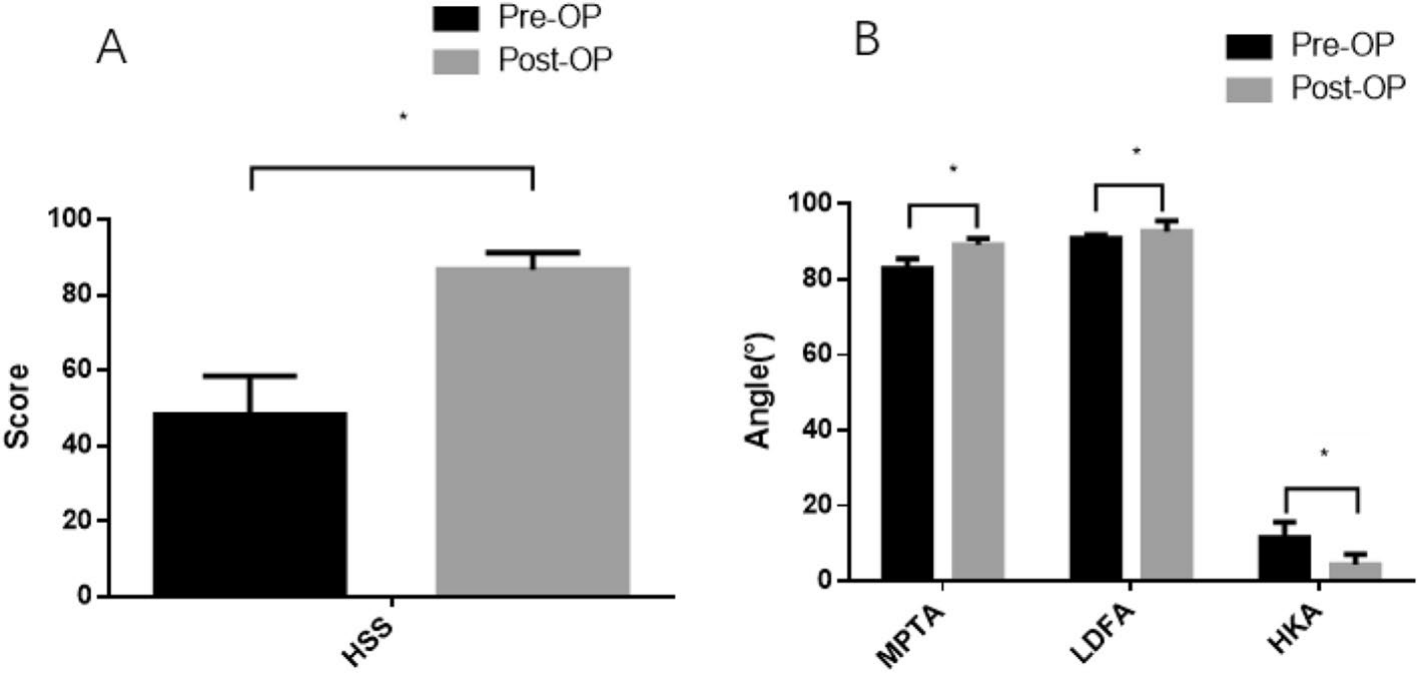
Statistic differences between preoperative and post-operative HSS score, MPTA, LDFA, HKA angles, respectively.

There was no significant difference in baseline characteristics regarding age, BMI and Follow-up time, which are shown in Table 1. For all patients, there are significant differences between pre-operative and post-operative HSS score, MPTA, LDFA, HKA angles, respectively. (Fig. 3A,B). In general, the HSS score were improved from 48.23 ± 10.34 points before surgery to 86.71 ± 4.49 points at the final follow-up examination (*p* < 0.05)**.**

**Table 1:** Pre-operative baseline characteristics for all patients, grouped according to post-operative mechanical alignment.

Group	NMA Group (n = 30)	VMA Group(n = 21)	*p*-value
Age	62.67 ± 5.72	64.13 ± 5.38	0.591
BMI	28.13 ± 3.19	27.42 ± 2.95	0.297
Pre-operative ROM	78.10 ± 4.02	78.83 ± 4.09	0.627
Pre-operative HSS	49.62 ± 10.07	47.27 ± 10.59	0.539

The values are given as the mean and the standard deviation.

*NMA group* neutral mechanical axis, *VMA group* varus mechanical alignment, *ROM* range of motion, *BMI* body mass index, *HSS* hospital for special surgery.

### Pre-operative and post-operative comparison between two groups

**Figure 4:**
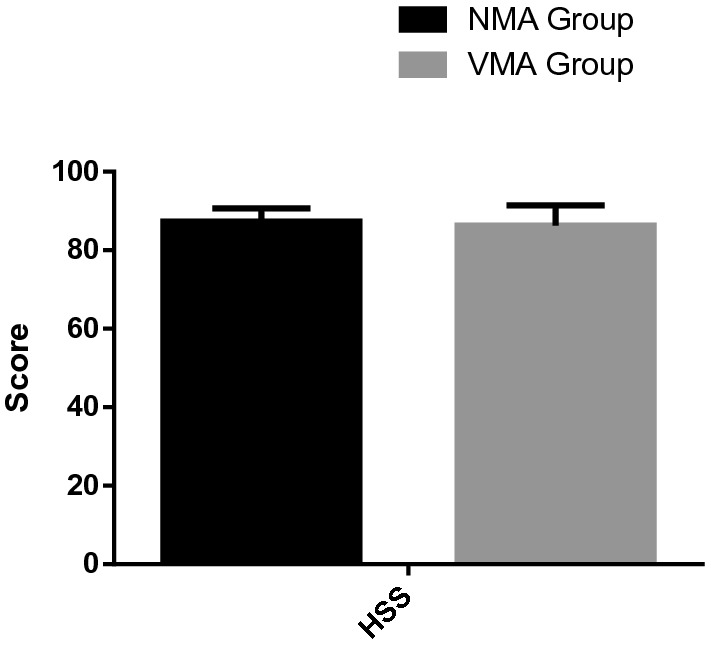
The difference of HSS score between the two groups after TKA.

Post-operative LDFA in NMA group was larger than VMA group (90.87° ± 2.13° vs 94.06° ± 2.60°), there was a significant statistical difference between the two groups (*p* = 0.001). Meanwhile, Post-operative MPTA in NMA group was larger than VMA group (89.81° ± 1.54° vs 88.78° ± 1.79°), there was a significant difference between the two groups (*p* = 0.038). For NMA group, the Mean and standard deviation of HKA was 1.80° ± 1.03°, for the VMA group, the Mean and standard deviation (11.53° ± 4.15°) were more than three and two times larger than those of NMA group. A significant difference was detected between the two groups (*p* < 0.05). No significant differences were observed in HSS score between NMA group and VMA group (87.29 ± 3.41 vs 86.30 ± 5.13) after operation (p < 0.05) (Fig. 4).

## Discussion

Previous studies^14,15^ showed inconsistent results, a retrospective study was conducted to better identify the association between mechanical alignment and post-operative HSS score after primary TKA in this study. The most important finding of the current study was that we found no significant association between post-operative lower limb mechanical alignment and HSS score for patients in Chinese with pre-operative constitutional varus knee caused by combined deformities. .

Experimental results on the association between mechanical alignment and HSS score in this research is consistent with previous studies ^15,22,25,26^. Michael et al^22^ proved that leaving varus knees in residual varus will not improve clinical outcomes in a retrospective review of 361 primary TKAs. Dominique et al. ^27^ also found there were similar clinical outcomes for neutral or varus alignment. Richard et al.^26^ concluded that there was no correlation between post-operative lower limb mechanical alignment and clinical outcomes for patients with pre-operative varus deformity. However, the previous research objects were Europeans or Americans who previously had varus knees. Here, the patients in Chinese with pre-operative constitutional varus knee caused by combined deformities were selected, and a new evidence was provided in this study.

For these OA patients with constitutional varus knee, there are many intraoperative difficulties and post-operative complications if applying neutral mechanical axis method. Firstly, it widely needs to release medial soft tissue, including socket stripping insertion of medial soft tissue of the tibia^17^, stripping popliteal ligament, superficial medial collateral ligament and so on^18^. It is widely known that excessive release of the soft tissue may result in bleeding, knee instability. Secondly, by applying reduction osteotomy of tibial plateau, Dixon et al.^19^ corrected severe varus knee deformity and improved patients' Knee Society Clinical Rating System (KSS) score and activity level. However, along with the narrowing of the tibial plateau, the contact area of the tibial plateau is decreased, meanwhile the wear of artificial joints will be increased under the same loading circumstance. Moreover, tibial prosthesis cannot cover the lateral tibial margin after reduction osteotomy, which will increase the sinking risk of the tibial component, because spongy bone of lateral-medial tibial plateau resulted in a relative ingression to adapt tracking of femoral component. When there exists residual varus deformity during the operation, the above complications can be reduced. In addition, obtaining neutral mechanical axis among these patients is more difficult and time-consuming procedure, which requires more complex bone cuts and larger soft tissue releases. Sampath et al.^28^ also described the need for increased operative time to obtain neutral axis of the lower limb in severe varus knees. In this study, slight varus mechanical alignment of the lower limb after TKA can reduce the difficulty and save operative time for surgeon.

This study has several limitations. Firstly, this study was a retrospective. Second, in the case of proximal tibia and distal femur bone on bone, the parameters of normal knee joint was measured. However, according to Dargel’s result^29^, there was a positive correlation in morphometric data between two knees for one subject. At last, static lower limb mechanical axis was not associated with clinical results while dynamic axis may be a predictor of clinical performances after TKA, because Miller et al.^30^ found the standing mechanical axis after TKA was not enough to predict dynamic behavior of the lower limb during gait. Thus, a prospective study with large samples is required in the future.

## Conclusion

For patients suffering from constitutional varus knee that caused by combined deformities among Chinese populations, we found no significant association between post-operative lower limb mechanical alignment and HSS score in our study. Slight varus mechanical alignment of the lower limb after TKA can reduce the difficulty and save operative time for surgeon.
